# A New Method to Isolate and Culture Rat Kupffer Cells

**DOI:** 10.1371/journal.pone.0070832

**Published:** 2013-08-14

**Authors:** Wei-qun Zeng, Ji-qin Zhang, Yue Li, Kang Yang, Yu-pei Chen, Zuo-Jin Liu

**Affiliations:** 1 Hepatobiliary Surgery Department, Second Affiliated Hospital of Chongqing Medical University, Chongqing, China; 2 Anesthesia Department, Second Affiliated Hospital of Chongqing Medical University, Chongqing, China; University College London, United Kingdom

## Abstract

**Background:**

Previous methods for Kupffer cells (KCs) isolation require sophisticated skills and tedious procedures. Few studies have attempted to explore the self-renewal capacity of KCs in vitro. Therefore, the aim of this study was to establish a simple method for rat KCs isolation and further investigate the mitotic potential of KCs in vitro.

**Methods:**

KCs were obtained by performing one-step perfusion, enzymatic tissue treatment, differential centrifugation and selective adherence. The proliferation ability of cultured KCs was determined by MTT assay and Propidium Iodide FACS analysis. Phagocytic assay and ED-1, ED-2 immunofluorescence were used to identify cell phenotype. After stimulation with LPS, the expression of surface antigens (MHCII, CD40, CD80, and CD86) and the production of cytokines (NF-κB, TNF-α, IL-6 and IL-10) were measured for cell function identification.

**Results:**

KCs were isolated with certain numbers and reasonable purities. The KCs were able to survive until at least passage 5 (P5), and at P3 showed equally strong phagocytic activity as primary KCs (P0). After stimulation with LPS, the change in the expression of surface antigens and the production of cytokines for P3 cells was similar to that for P0 cells.

**Conclusions:**

Our study provides a simple and efficient method for KCs isolation, and reveals that self-renewing KCs have the same phagocytic activity and functions as primary KCs.

## Introduction

Kupffer cells (KCs), named after the pathologist C. von Kupffer, are resident hepatic macrophages that account for 80–90% of total fixed tissue macrophages in the body [1]. An important physiological function of these cells is their ability to eliminate and detoxify microorganisms, endotoxins, degenerated cells, immune complexes, and toxic agents [2]. Therefore, KCs play an important role in liver physiological homeostasis and are intimately involved in the liver's response to infection, toxins, transient ischemia, and various other stresses [3] through the expression and secretion of soluble inflammatory mediators [4], [5]. KCs can be classically activated (M1) or alternatively activated (M2) [6]. M1 macrophages are associated with the proinflammatory response and produce associated cytokines such as IL-1b, IL-12, IL-23, and TNF-a. M2 macrophages are associated with downregulation of immune responses [7] and IL-10 production [8]. Cytokines act as protective mediators for recovery of normal liver function [9], however, in some instances, excessive activation of KCs may result in exacerbation of the damage [10]. Proper therapeutic modulation of the inflammatory activities of KCs provides opportunities for new treatment approaches toward liver disease, and primary cell culture is indispensable for further studies in this area.

Most previous methods of isolating KCs included two-step collagenase-pronase perfusion followed by gradient centrifugation [11], [12]. Although these methods provide certain numbers of KCs with reasonable purity, they require sophisticated skills, equipment and tedious cell isolation procedures. Furthermore, although the expansion of KCs has been demonstrated by zymosan stimulation, recombinant GM-CSF stimulation or two-thirds partial hepatectomy experimental models [13]–[15], few studies have attempted to demonstrate the self-renewal and subculture ability of normal KCs in vitro. Therefore, the aims of this study are to establish a simple and efficient method to isolate KCs as well as further investigate the mitotic potential of normal KCs in vitro.

## Materials and Methods

### Ethics Statement

All of the animal procedures were approved by the Committee on the Ethics of Animal Experiments of Chongqing Medical University. This investigation was carried out in strict accordance with recommendations in the Guide for the Care and Use of Laboratory Animals of the National Institutes of Health. All of the surgeries were performed under anesthesia, and all efforts were made to minimize suffering.

### Isolation and Culture of KCs

Adult male Sprague-Dawley rats (8 to 10 weeks old, 200 to 250 g) were obtained from the experimental animal center of Chongqing Medical University. The animals were anesthetized by the inhalation of ether and placed in a supine position on a plastic tray. The abdomen was opened to confirm the location of the portal vein, and the thoracic cavity was opened to expose the heart. A cut was made in the right atrium wall, simultaneously, the portal vein was cannulated with a 22G catheter and the liver was perfused in situ with phosphate-buffered saline (PBS) at a rate of 10 ml/min for 5 min. The liver was then excised, transferred into a sterile beaker and washed twice with PBS.

The organ was minced to small pieces and digested using 30 ml of 1 mg/mL collagenase type IV (Sigma, USA) at 37°C. After incubation in a water bath for 30 min, the liver homogenate was filtered through a cell strainer (100 µm) to remove undigested tissue fragments. The filtrate was transferred into 50 ml conical tubes and centrifuged twice at 300×g (4°C) for 5 min to wash out the residual enzymatic solution. The supernatant was discarded, the pellet resuspended and differential centrifugation was performed to separate non-parenchymal from parenchymal cells. After centrifuged the cell suspension at 50×g (4°C) for 3 min, the supernatant was placed into another 50 ml conical tube. Finally, the cell suspension was centrifuged at 300×g for 5 min and the supernatant was discarded.

The cell pellet was seeded on six-well culture plates in complete culture medium (Dulbecco's Modified Eagle's Medium (DMEM, Hyclone, USA) supplemented with 10% fetal bovine serum (FBS, Gibco, Aus), 100 µg/ml streptomycin and 100 U/ml penicillin). Following incubation for 2 h in a humidified atmosphere of 95% air with 5% carbon dioxide (CO_2_) at 37°C, the cells were gently washed with fresh culture medium. The medium was then changed every 2–3 days and the cells were cultured as passage 0 (P0). During the following cultivation period, the cells were washed with PBS after reaching 80% confluence and subsequently detached by incubation with 0.25% trypsin, counted using a hemocytometer and sub-cultured in new culture plates (P1). The same conditions were used for subsequent passages (P2–5).

### Immunofluorescence

P0 and P3 cells were seeded in 24-well chamber slides at a density of 2×10^4^ cells per well with the culture medium, respectively. After 1 day of culture, the slides were rinsedwith PBS and fixed with 4% paraformaldehyde at room temperature for 15 min. After permeabilization with 0.1% Triton X-100 for 10 min followed by blocking with 10% normal goat serum for 30 min, the cells were incubated with the following antibodies overnight at 4°C: rabbit polyclonal anti-CD68 (ED-1, Bioss, China), rabbit polyclonal anti-CD163 (ED-2, Bioss, China), rabbit polyclonal anti-CK18 (Bioss, China), rabbit polyclonal anti-CD31 (Bioss, China) and rabbit polyclonal anti- alpha-SMA (Santa Cruz, USA). To exclude a-specific binding the primary antibody was omitted and incubated solely with secondary antibody. The slides were rinsed with PBS three times and incubated with goat anti-rabbit IgG labeled with fluorescein isothiocyanate (FITC) away from light. After incubation for 1 h at room temperature, the cells were washed three times with PBS and the slides were covered with mounting medium containing DAPI for 10 min in a dark environment. The immunostained slides were observed and photographed by a fluorescent microscope equipped with digital camera system (Olympus).

### Phagocytic Assay

The isolated cells were seeded in two 60 mm culture dishes (one dish for P0 and the other for P3) at a density of 1×10^5^ cells per dish with the culture medium and incubated with sterile India ink at a dilution of 1∶100 for 4 h at 37°C. After incubation, the cells were rinsed with PBS three times to remove nonphagocytosed ink and photographed using a phase contrast microscope with digital camera (Olympus).

For the visualization of LDL and latex beads uptake, P0 and P3 cells were incubated with Dil -LDL 5 µg/ml (Invitrogen, USA) and latex beads at a ratio of 10 beads per cell (0.5 µm diameter) (Sigma, USA) for 4 h at 37°C, respectively. After incubation, the cells were gently washed three times with sterile PBS and imaged by fluorescent microscopy.

### Cell Proliferation

Cell proliferation ability was determined using the MTT assay [16]. P0 cells were seeded in 96-well plates in triplicate at a density of 5×10^3^ cells per well with the complete culture medium. A total of 7 plates were plated. The culture medium was replaced every 2–3 days. Every two days, the cells in one plate were washed with PBS solution, 180 µl DMEM (without FBS) and 20 µl of MTT solution (final concentration 0.5 mg/ml) was added into each well. After incubation for 4 h, the MTT solution was removed and 150 µl of dimethyl sulfoxide (DMSO) was added to each well. The plate was shaken for 10 min. The formation of purple formazan crystals, which are proportional to the number of metabolically active viable cells, was determined by measuring the optical density at 490 nm wavelength on a microplate reader.

### Flow Cytometry

P0 and P3 cells were seeded in two 60 mm culture dishes at a density of 1×10^6^ cells per dish, respectively. The next day, the medium was replaced with culture medium containing LPS at 1 µg/ml. After incubation for 24 h at 37°C, the cells were detached by incubation with 0.25% trypsin and pelleted by centrifugation for 5 minutes at 800 r/min. The cells were resuspended in 1 ml PBS, and anti-rat MHCII-FITC, CD40-FITC, CD80- phycoerythrin(PE), and CD86-PE antibodies (Ebioscience, USA) were added according to the manufacturer's instructions. After incubation in a dark environment for 30 min at room temperature, cell fluorescence was evaluated by flow cytometry (BD Biosciences, USA).

### Cytokine Production

P0 and P3 cells were seeded in six-well culture plates at a density of 2×10^5^ cells per well, respectively. The next day, the medium was replaced by the fresh culture medium and the cells were cultured in the presence of LPS at 1 µg/ml. After incubation for 0 h and 7 h at 37°C [17], [18], the culture supernatant was collected and stored at −20°C until measured. Standard enzyme-linked immunoabsorbent assay (ELISA) kits (USCN, China) were used to determine levels of TNF-α, NF-κB, IL-6 and IL-10 in the culture media, according to the manufacturer's instructions. The experiments were performed independently four times, and cytokine concentrations in the culture supernatant are expressed as the mean values ± standard deviation (SD).

### Statistical Analysis

Data are expressed as mean values ± SD. The statistical significance of differences between mean values was determined by factorial analysis. A *P* value of less than 0.05 was considered to be significant. Statistical calculations were performed using SPSSv18 software (SPSS Inc., Chicago, Illinois, USA).

## Results

### Primary Culture and Sub-culture of KCs

**Figure 1:**
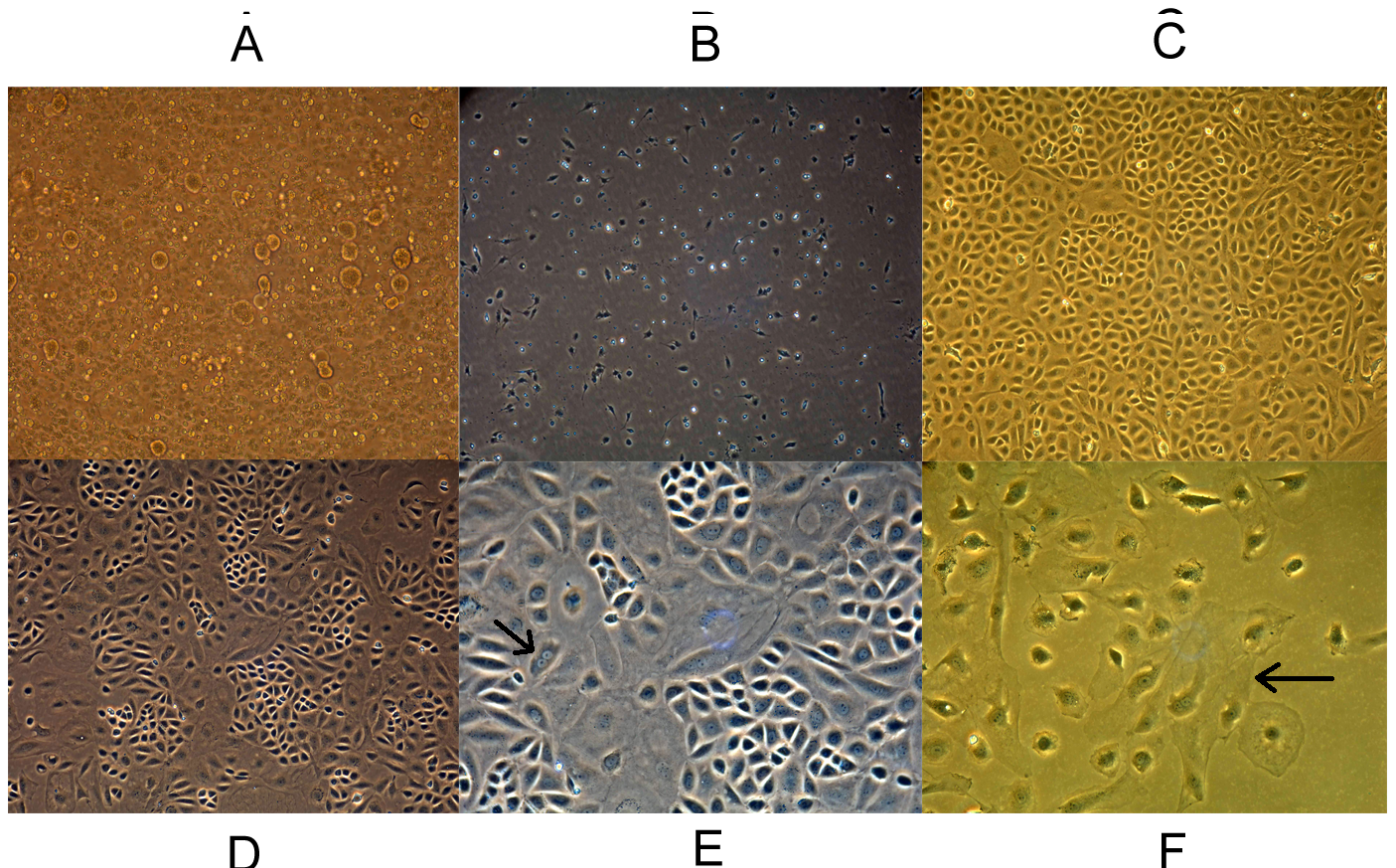
Photomicrographs of Kupffer cells (KCs) in culture (100×). (**A**) Non-parenchymal hepatocyte-rich cell fractions. (**B**) Primary KCs, after incubation for 2 h. (**C**) KCs after culturing at maximum levels. (**D**) KCs cultured as passage 5. (**E**) Cells in mitosis (arrow). (**F**) Multinuclear giant cells (arrow).

**Figure 2:**
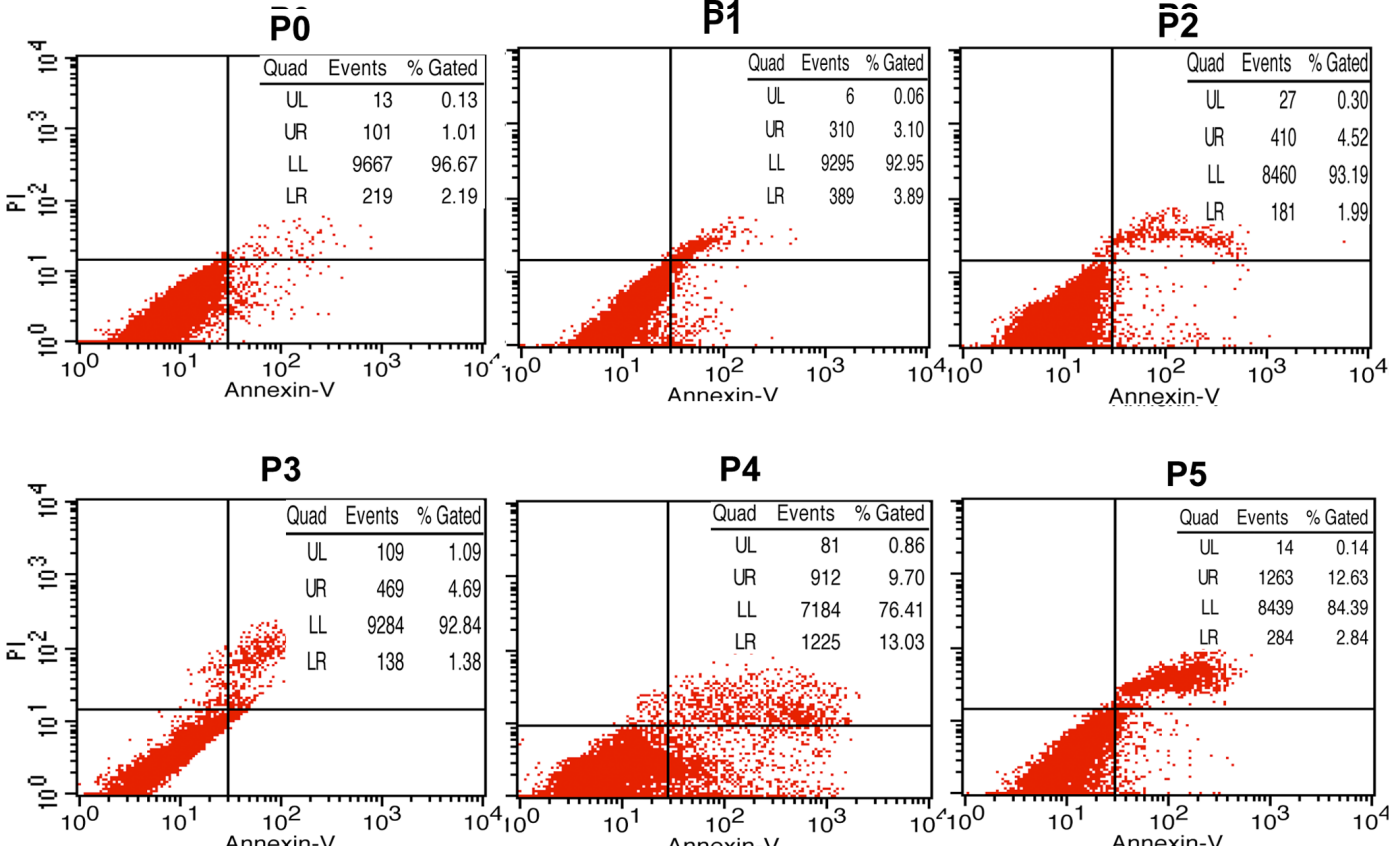
Apoptosis tests of primary KCs and subcultured cells. The presence of apoptotic cells was identified by flow cytometric analysis of cells labeled with Annexin V and propidium iodide. Cells in the lower right quadrant correspond to early apoptotic cells (Annexin V-positive and propidium iodide-negative), while cells in the upper quadrant correspond to late apoptotic or necrotic cells (Annexin V-positive and propidium iodide-positive). The results showed that the percentage of apoptotic cells was increased over the course of passaging (especially after passage 3).

The sinusoids of the liver after the perfusion appeared patent and free of circulating blood cells. In vitro enzymatic digestion of liver tissue is perhaps the most crucial step of cell isolation as the final cell yield closely depends on the extent of tissue dissociation. After differential centrifugation, non-parenchymal hepatocyte-rich cell fractions were obtained and cultured in tissue culture plates (P0) (Fig. 1A). After incubation for 2 h, the cells attached to the plate surface. The plates were rinsed with PBS to remove non-adherent cells, and attached KCs were selectively harvested with high purity (90%) (Fig. 1B). The average yield was more than 10^7^ cells and the viability of cells (95%) was determined by trypan blue exclusion. The cells exhibited round nuclei with an irregular outline, which resembles to the shape of resting macrophages in culture. The cultured KCs could be harvested as early as day 8, and numbers reached maximal levels on days 12 to 14, when the cells formed a flat cell sheet on the plate surface (Fig. 1C). After colonies of KCs reached 80% confluence, the cells were treated with 0.25% trypsin and sub-cultured in new culture plates (P1). All of the obtained KCs survived until P5 (Fig. 1D). However, apoptosis tests (double staining of Annexin V and propidium iodide) showed that the percentage of apoptotic cells increased over the course of passaging (especially after passage 3) (Fig. 2).

To our knowledge, this is the first study reporting proliferation of ordinary KCs (without any stimulation) and the subculturing potentials of the cell sheet of mixed primary cultures of rat liver cells. During cell culture, cells in mitosis (Fig. 1E, arrow) and multinuclear giant cells were occasionally observed under a phase contrast microscope (Fig. 1F, arrow).

### P0 and P3 Cells Stained Strongly Positive for ED-1/ED-2 and Similar Phagocytic Activity

**Figure 3:**
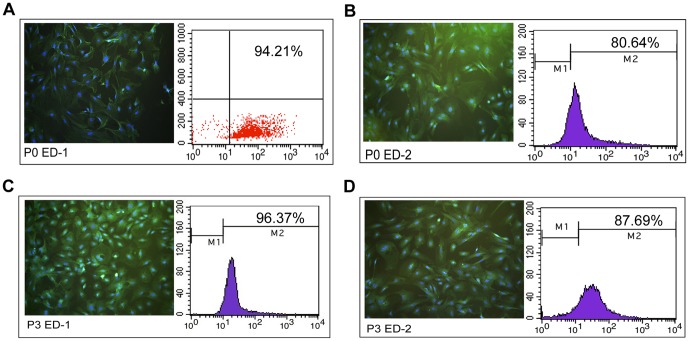
The positive ratios of ED-1/ED-2 in P0 and P3 (100×). The positive ratios for ED-1 in P0 and P3 cells were 94.21% (**A**) and 96.37% (**C**), respectively, while the ratio for ED-2 in P0 and P3 cells were 80.64% (**B**) and 87.69% (**D**), respectively. (Blue: DAPI, Green: FITC beads).

**Figure 4:**
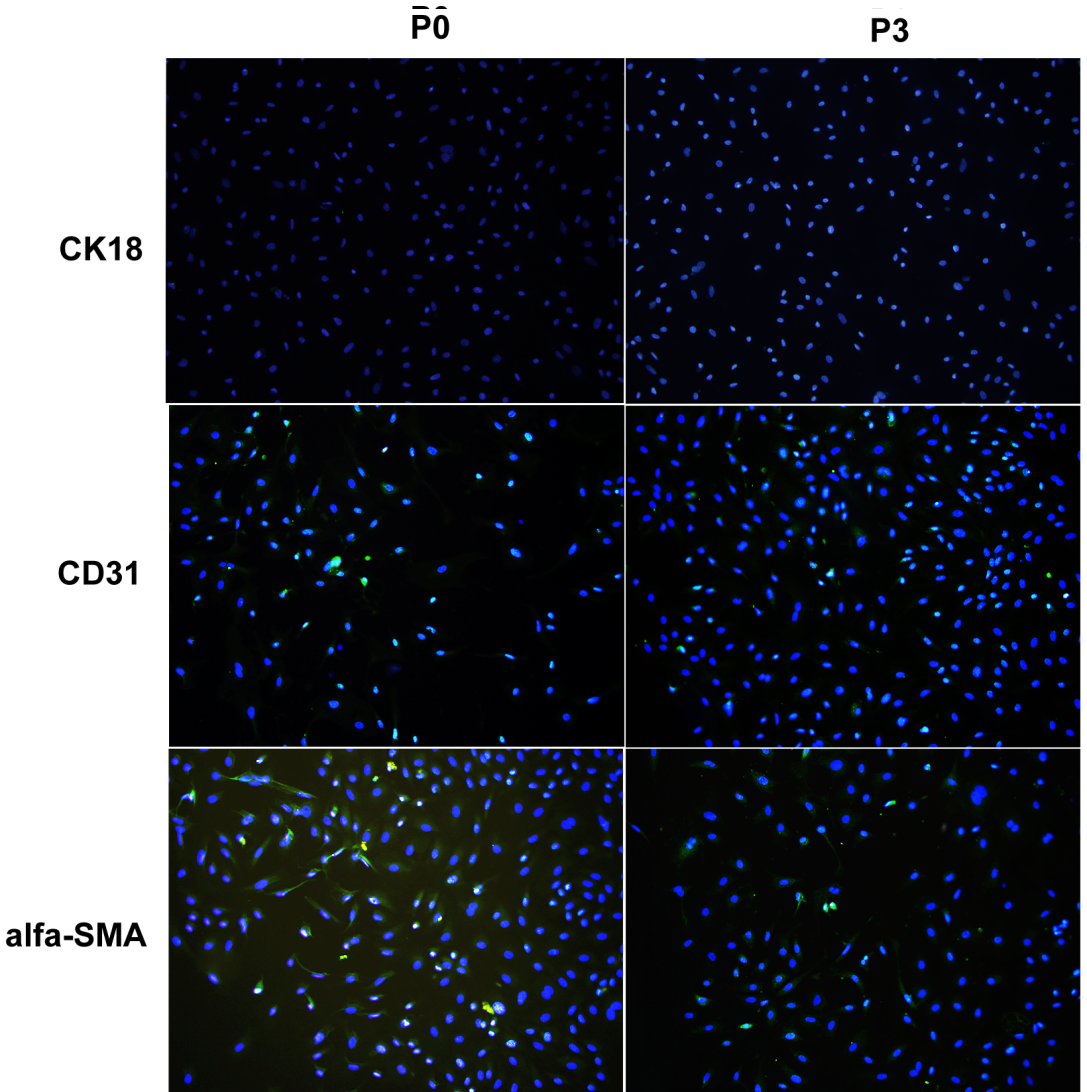
Immunofluorescence tests for contaminating cells (100×). The results indicated that contaminating cells detected both in P0 and P3 cell cultures were few and included SECs (positive for CD31) and HSC (positive for alpha-SMA), but not hepatocytes (positive for CK18). (Blue: DAPI, Green: FITC beads).

ED-1 and ED-2 are the markers of rat macrophages [19], and it has been reported that ED-2 immunocytochemistry is more specific and allows for discrimination between KCs and monocytes recently recruited in the liver tissue [20]. P0 and P3 cells were incubated with rabbit polyclonal anti-ED1 and anti-ED2. After incubation with goat anti-rabbit IgG labeled with FITC and DAPI, the immunostained slides were observed and photographed by a fluorescent microscope. Both P0 and P3 were strongly positive for ED-1 and ED-2. In addition, FACS analysis demonstrated the positive ratios for ED-1 in P0 and P3 cells were 94.21% (Fig. 3A) and 96.37% (Fig. 3C), respectively, and ED-2 in P0 and P3 cells were 80.64% (Fig. 3B) and 87.69% (Fig. 3D), respectively. In addition, immunofluorescence tests indicated that few contaminating cells were detected in either the P0 or P3 cell cultures. These contaminating cells included SECs (positive for CD31) and HSC (positive for alpha-SMA), but not hepatocytes (positive for CK18) (Fig. 4).

**Figure 5:**
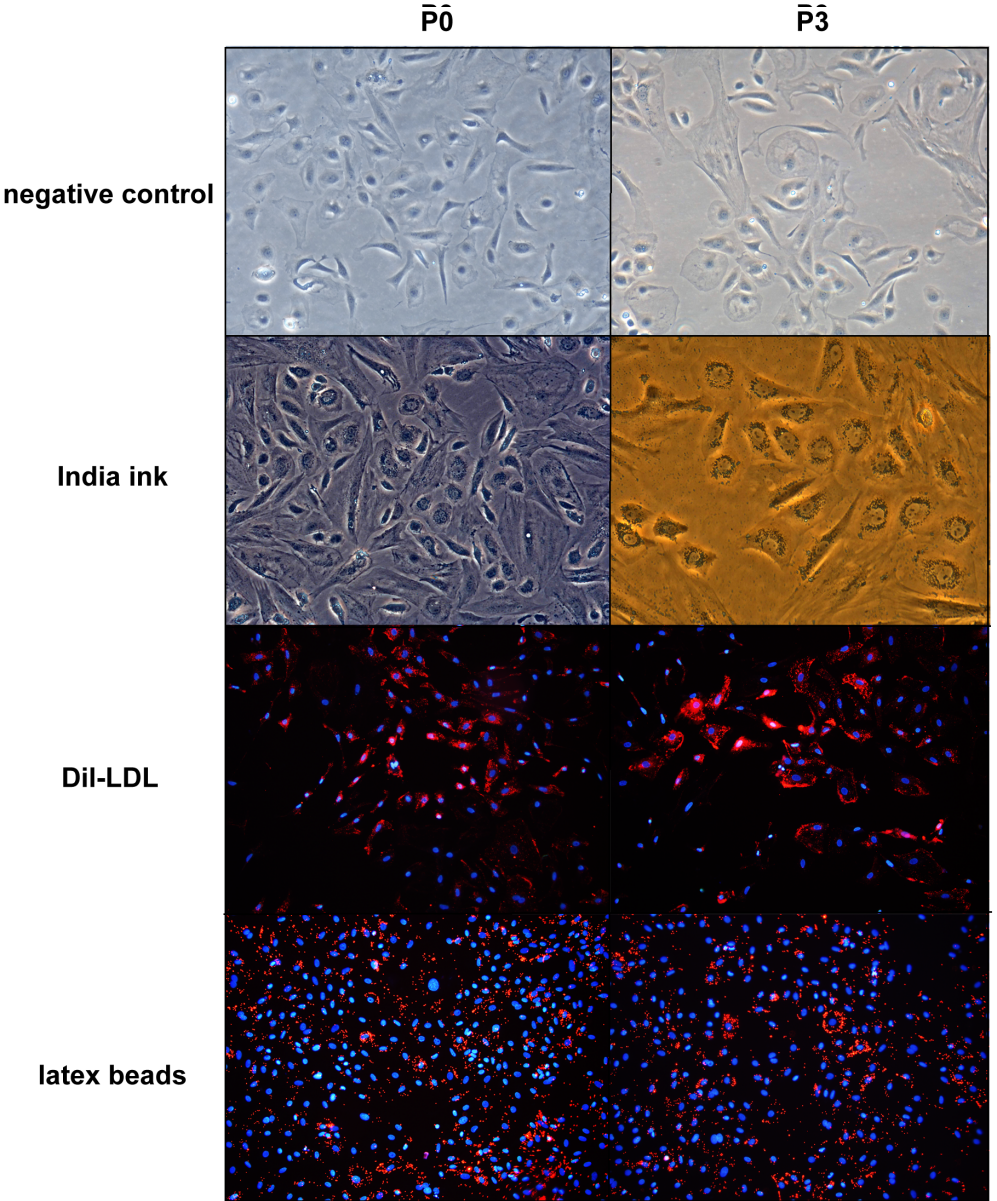
Phagocytic activity of kupffer cells (100×). Both P0 and P3 cells displayed strong phagocytic activity: nearly all of the cells incorporated the ink, Dil-LDL and latex beads 4 h after administration. In addition, there were no significant differences in the phagocytic abilities of P0 and P3.

Phagocytosis of India ink by both P0 and P3 cells were analyzed with a phase contrast microscope and the uptake of Dil-LDL and latex beads were imaged by fluorescent microscopy. As it shown, both P0 and P3, nearly all of the cells incorporated the ink, Dil-LDL and latex beads 4 h after administration. These results demonstrate strong phagocytic activity, a functional characteristic of KCs, by both P0 and P3 cells (Fig. 5).

### Kinetics of KCs Proliferation

**Figure 6:**
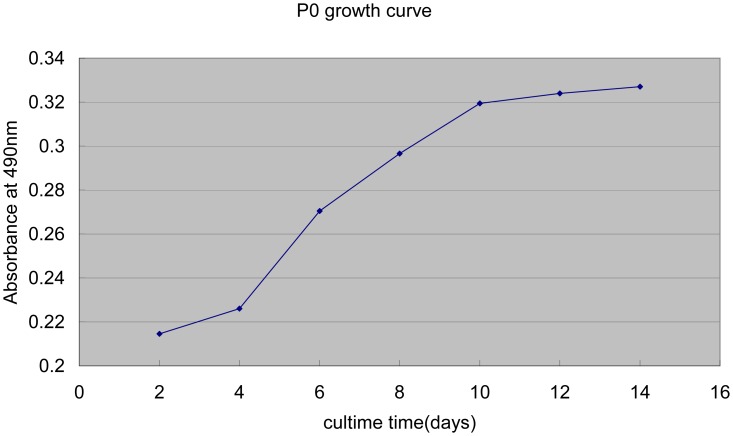
Growth curve of primary KCs. The growth curve revealed an “S” shape. The detention period, in which the proliferation speed of cells was slow, was between 2–4 days. The exponential phase of growth, meaning the cells rapidly proliferated, was 4–10 days, and the following 4 days were the platform period, when the cells achieved saturation density. The data are expressed as mean values obtained by three determinations.

**Figure 7:**
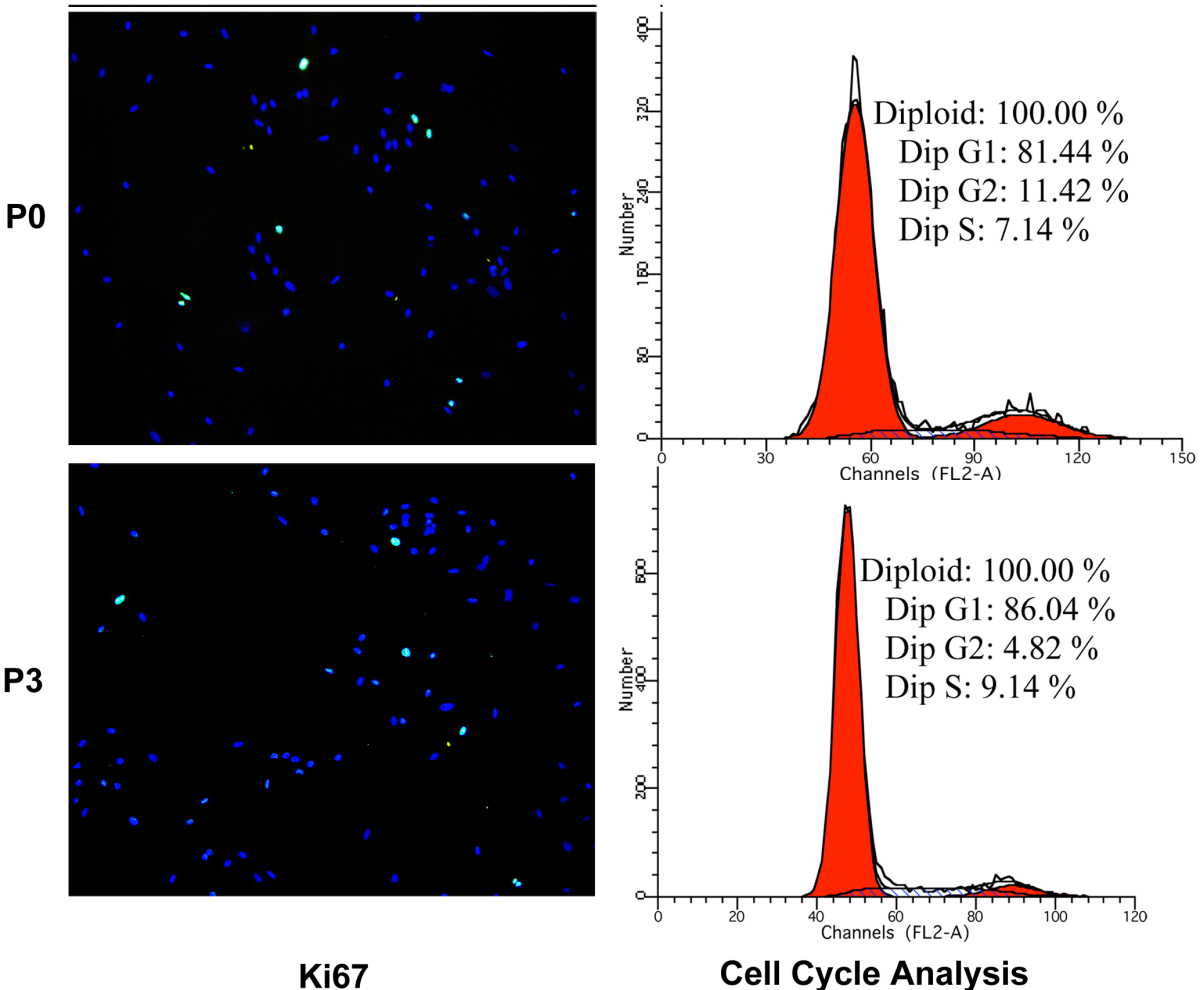
Immune-fluorescence tests for Ki67 (100×) and Propidium Iodide FACS analysis. P0 and P3 were harvested following 8 days of culture. Ki67 was used as a marker for cell proliferation. In a process known as cycle analysis, propidium Iodide FACS analysis was used to measure the DNA content of cells and distinguish between cells in G1, S, and G2/M. The results showed that P0 and P3 cells were positive for Ki67 and there were a large number of cells in S and G2/M, confirming the existence of proliferation phenomena in P0 and P3. (Blue: DAPI, Green: FITC beads).

After cell inoculation at 2, 4, 6, 8, 10, 12 and 14 days, the optical density of purple formazan crystals was measured. The growth curve (Fig. 6) of KCs showed an “S” shape: 2–4 days was the detention period, in which the proliferation speed of cells was slow; Four to ten days was the exponential phase of growth, meaning that the cells rapidly proliferated; Following 4 days was the platform period, when cells achieved saturation density. Furthermore, immune-fluorescence of Ki67 (a proliferation marker [21]) and Propidium Iodide FACS analysis (cell cycle analysis) confirmed the existence of proliferation phenomenon (Fig. 7). To our knowledge, this is the first study reporting the kinetics of KCs proliferation under normal physiological conditions.

### P0 and P3 Cell Functions Had No Significant Differences

**Figure 8:**
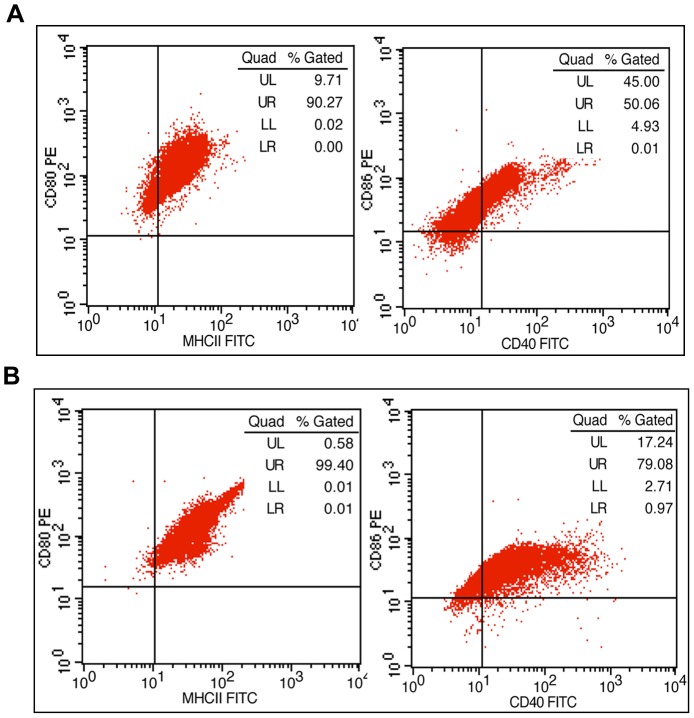
The expression of surface antigens. (**A**) P0. (**B**) P3. The positive ratios of MHCII (99.40%) and CD40 (80.05%) for P3 cells were higher than the ratio for P0 (MHCII: 90.27%; CD40: 50.07%); the positive ratios for CD86 in P0 and P3 cells were over 95%; the CD80 positive ratios in P0 and P3 cells were both approximately 99%.

**Figure 9:**
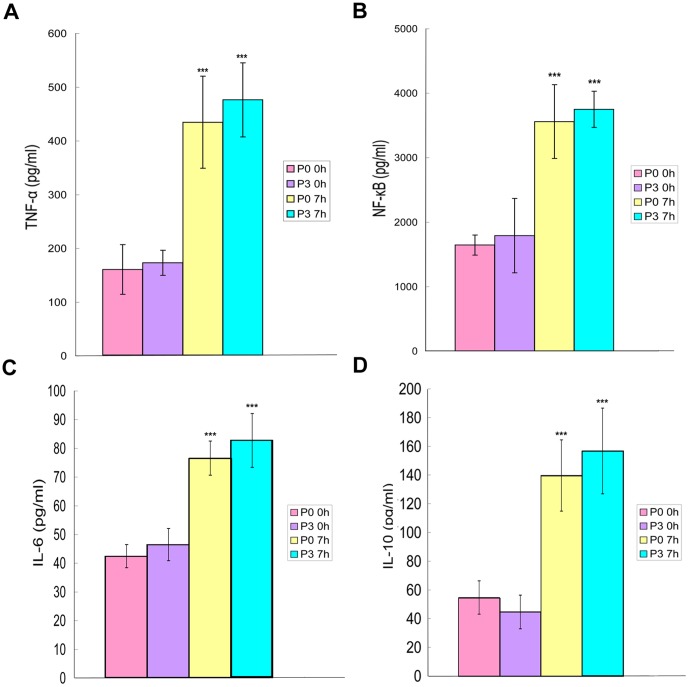
Cytokine production. P0 and P3 cells were stimulated with LPS for 0 h and 7 h. The abundance of TNF-α (Fig. 9A), NF-κB (Fig. 9B), IL-6 (Fig. 9C) and IL-10 (Fig. 9D) increased over time and showed significant differences (*p*<0.001). However, there were no significant differences detected between P0 and P3 cells for inflammatory or anti-inflammatory cytokines at any of the time points measured. Error bars represent mean values ± standard deviation; *** *p*<0.001.

To evaluate the functional capacity of P0 and P3, the expression of the surface antigens (MHCII, CD40, CD80, and CD86) and the production of cytokines (NF-κB and TNF-α) were measured. Among the four antigens examined, the positive ratios of MHCII (90% or over) and CD40 (80% or over) in P3 cells (Fig. 8B) were higher than those in P0 cells (Fig. 8A). The positive ratios for CD86 in P0 and P3 cells were over 95%, and the CD80 positive ratios in P0 and P3 cells were all approximately 99%. After stimulation with LPS for 7 h, P0 and P3 cells secreted significant amounts of inflammatory cytokines (TNF-α, NF-κB, IL-6) and anti-inflammatory cytokines (IL-10). In both P0 and P3 cells, the abundance of TNF-α (Fig. 9A), NF-κB (Fig. 9B), IL-6 (Fig. 9C) and IL-10 (Fig. 9D) increased over time and were significantly different (*p*<0.001). However, no significant differences were detected between P0 and P3 cells with respect to inflammatory or anti-inflammatory cytokines at any of the time points measured.

## Discussion

Most previous methods for KCs isolation utilized two-step collagenase perfusion in situ [22] which were skillful and needed large amount of collagenase. The liver tissue often treated with pronase to eliminate parenchymal hepatocytes [23], however, pronase may destroys the lipopolysaccharide receptor CD14 on KCs [24]. Finally, obtained cells by percoll density gradient differential centrifugation [25], though the procedures are sophisticated and tedious (the time of centrifugation was more than 40 min in previous methods, while we needed fewer than 20 min). In our protocol, we utilized one-step PBS perfusion in situ, the main purpose of which was to free the hepatic sinusoids from circulation blood cells. Therefore, one of the most critical steps was the enzymatic digestion (we used only collagenase, without pronase) of liver tissue by water bath in vitro. Over-digestion resulted in a low yield of viable cells, while under-digestion would made it difficult to separate the cells [26]. The liver is comprised of parenchymal hepatocytes and non-parenchymal cells [27], and density as well as cell size are significantly different between the cell types. Due to this fact, we obtained the non-parenchymal cell fraction via differential centrifugation and cultured it. To purify KCs, we used the method of selective adherence to plastic, which is one of KC's basic biological characteristics. Previous studies have demonstrated that hepatic non-parenchymal cells, such as sinusoidal endothelial cells (SECs), KCs and hepatic stellate cells (HSCs) [28], share certain biological characteristics, such as adhering to glass. However, the attachment time were significantly different (SECs: 3 h [29]; KCs: 2 h [30]; HSCs: 24 h [31]). After cultivation for 2 h, we rinsed the culture plates with PBS to eliminate possible contamination with other hepatic non-parenchymal cell types. Adherent KCs were selectively harvested with high purity (approximately 80–90% positive for ED-2), which is similar to Valatas' study using traditional methods [32].

The origin of KCs remains controversial. Certain studies indicated that KCs are derived from blood monocytes and precursor cells in bone marrow [33] and that, as a type of mature differentiated cells, are unable to divide. However, the expansion of KCs had been demonstrated by zymosan stimulation, recombinant GM-CSF and two-thirds partial hepatectomy experimental models [13], [15]. Consistent with the latter results, we also found that isolated KCs showed vigorous self-renewal and subculturing potential. Obviously, the self-renewal and subcultured capacity do not contribute to hepatic non-parenchymal cell contamination. Firstly, the subculture KCs presented the same cytochemical and functional characteristics as the primary KCs, including the exclusive localization of ink, LDL and latex beads, and the expression of ED-1 and ED-2. Furthermore, the expression of the surface antigens (MHCII, CD40, CD80, and CD86) and the production of cytokines (NF-κB, TNF-α, IL-6, IL-10) of passaged cells were similar to or even superior to those of the primary KCs after stimulation with LPS. In addition, the most common mixed cells in primary cultures of KCs, such as hepatocytes and HSC, especially hepatocytes, survive and maintain their morphological and physiological properties for only a limited duration. During apoptosis of these cells or as the cells undergo a phenotypic and functional conversion, which is referred to as an epithelial-mesenchymal transition (EMT), superfluous fibroblastic cells emerge [34]. Studies suggest the pathogenesis of fibrosis is tightly regulated by macrophages that exert unique functional activities throughout the initiation, maintenance, and resolution phases of fibrosis [35]. Therefore, it is possible that the proliferation of KCs is a response to the culture environmental changes in the culture caused by transformed hepatocytes and/or HSC during EMT.

In conclusion, we have established a simple and efficient method to isolate KCs from mixed primary cultures of rat liver cells. Most interestingly, our isolated KCs showed vigorous self-renewal and subcultured capacity during their normal steady state.
